# Submaximal Exercise Provokes Increased Activation of the Anterior Default Mode Network During the Resting State as a Biomarker of Postexertional Malaise in Myalgic Encephalomyelitis/Chronic Fatigue Syndrome

**DOI:** 10.3389/fnins.2021.748426

**Published:** 2021-12-15

**Authors:** Rakib U. Rayhan, James N. Baraniuk

**Affiliations:** ^1^Department of Physiology and Biophysics, Howard University, Washington, DC, United States; ^2^Department of Medicine, Georgetown University, Washington, DC, United States

**Keywords:** postexertional malaise, default mode network, DMN, blood oxygenation level dependent, BOLD, fibromyalgia, chronic idiopathic fatigue, submaximal exercise

## Abstract

**Background:** Myalgic encephalomyelitis/chronic fatigue syndrome (ME/CFS) is characterized by disabling fatigue and postexertional malaise. We developed a provocation paradigm with two submaximal bicycle exercise stress tests on consecutive days bracketed by magnetic resonance imaging, orthostatic intolerance, and symptom assessments before and after exercise in order to induce objective changes of exercise induced symptom exacerbation and cognitive dysfunction.

**Method:** Blood oxygenation level dependent (BOLD) scans were performed while at rest on the preexercise and postexercise days in 34 ME/CFS and 24 control subjects. Seed regions from the FSL data library with significant BOLD signals were nodes that clustered into networks using independent component analysis. Differences in signal amplitudes between groups on pre- and post-exercise days were determined by general linear model and ANOVA.

**Results:** The most striking exercise-induced effect in ME/CFS was the increased spontaneous activity in the medial prefrontal cortex that is the anterior node of the Default Mode Network (DMN). In contrast, this region had decreased activation for controls. Overall, controls had higher BOLD signals suggesting reduced global cerebral blood flow in ME/CFS.

**Conclusion:** The dynamic increase in activation of the anterior DMN node after exercise may be a biomarker of postexertional malaise and symptom exacerbation in CFS. The specificity of this postexertional finding in ME/CFS can now be assessed by comparison to post-COVID fatigue, Gulf War Illness, fibromyalgia, chronic idiopathic fatigue, and fatigue in systemic medical and psychiatric diseases.

## Introduction

Myalgic encephalomyelitis/chronic fatigue syndrome (ME/CFS, abbreviated here as CFS) has been defined by subjective symptom criteria. The 1994 Center for Disease Control (“Fukuda”) criteria required unrelenting fatigue for over 6 months with no medical or psychiatric explanation plus four of eight ancillary criteria: short term memory or concentration, sleep disturbances, post-exertional malaise (PEM), headache, myalgia, arthralgia, sore throat or sore lymph nodes (Fukuda et al., 1994). The criteria were improved by including autonomic and flu-like symptoms in the Canadian Consensus Criteria (Carruthers et al., 2003, 2011; Carruthers, 2007). The emphasis shifted to fatigue, PEM, and unrefreshing sleep with either cognitive dysfunction or orthostatic intolerance in the 2015 Institute of Medicine criteria for Systemic Exercise Intolerance Disease (SEID)(Institute of Medicine, 2015). The 2020 National Institute for Health and Care Excellence (NICE) guidance reinforced the core symptoms of fatigue, PEM, cognition, and sleep (National Institute for Health and Care Excellence [NICE], 2021).

Post-exertional malaise has been described as exertional exhaustion or postexertional symptom exacerbation. Performing modestly greater than usual physical, cognitive, emotional or other effort leads to a “collapse” and relapse of dysfunctional impairment with fatigue, flu-like malaise, widespread pain, tenderness, orthostatic intolerance, and cognitive dysfunction of attention and working memory. Onset may be immediate or delayed overnight. The consequences are out of proportion to the effort as subjects with PEM can become bed bound and unable to complete usual daily activities or productive work for several days or longer.

The characteristics of PEM were evaluated using a submaximal bicycle exercise provocation paradigm to provoke and quantify fatigue, cognition, autonomic control of postural tachycardia, and other processes that may contribute to this dynamic alteration. Subjects completed two submaximal bicycle exercise stress tests on consecutive days. We discovered the novel finding that submaximal exercise induced transient postural tachycardia in about 25% of study participants (Rayhan et al., 2013; Garner et al., 2018; Garner and Baraniuk, 2019) and as a result investigated exercise-induced orthostatic tachycardia as a potential contributor to cognitive dysfunction and PEM.

Functional magnetic resonance imaging was performed before the stress tests to identify baseline differences in blood oxygenation level dependent (BOLD) signals between groups that may be diagnostic, and again after provocation to identify exertion-induced alterations that may be markers of PEM (Rayhan et al., 2013, 2019; Garner et al., 2018; Garner and Baraniuk, 2019; Washington et al., 2020a, b). The current study evaluates the resting state (no task) and can be compared to scans during a simple 0-back task of attention, and a difficult 2-back verbal working memory task to assess changes related to increasing cognitive task load. Brain regions that were activated or deactivated were identified as nodes; their correlated, synchronous activation with other nodes defined large scale cognitive networks. Networks provide the integrated services needed to cognitively observe and evaluate the environment, plan tasks and marshal resources for task completion, then rest and contemplate the actions during periods with no externally directed activity.

Being at rest leads to activation of nodes in the Default Mode Network (DMN) that have increased spontaneous activity at rest or, alternatively, are activated “by default” when there is no task to perform (Esposito et al., 2006). This network is activated for inward reflective thought, planning and self - referential contemplation. The DMN is anatomically housed in the medial anterior prefrontal cortex (mPFC, DMN_mPFC), posterior cingulate cortex (PCC, DMN_PCC), precuneus (DMN_Prec), and lateral parietal and inferior parahippocampal nodes (Uddin et al., 2009). A remarkable feature of the BOLD analysis during rest is that traces of task, sensorimotor, auditory, visual, and other networks are also apparent (Fox et al., 2005). Task networks have roles for salience, attention, and executive control. The salience network (SAL) provides oversight of task activity by monitoring ongoing sensory and other stimuli through the bilateral anterior insulae (aINS) for higher level management by the dorsal anterior cingulate cortex (dACC). Focus on the task is maintained by surveillance of the Dorsal Attention Network (DAN) in the frontal eye fields of the rostral middle frontal gyri and bilateral intraparietal sulci. Executive control (EC) is maintained by bilateral dorsolateral prefrontal cortex (DLPFC) and parietal regions of the Frontal Parietal Network (FPN) that encodes task actions that are carried out by the sensorimotor network (SMN). Occipital nodes form the Visual network (VIS).

Local structural injury or molecular dysfunction of neurons in the cortex, oligodendrocytes in myelin sheaths of long fiber tracts, or supporting microglia and astrocytes can disrupt the coordination between nodes and compromise network activities during tasks and at rest. Mechanisms leading to atrophy or hypertrophy in one region can spread along axons in long white matter tracts to transmit these pathological alterations to other nodes in the interconnected network. The network denegation hypothesis (Vanasse et al., 2021) contends that pathological injury in one localized region can be transmitted to connected brain regions via networks, and suggests that dysfunction in one node will predict that other regions in the associated network will become preferentially affected. The concept of network dysfunction may also predict that diseases with different pathologies may have superficial similarities that can be resolved by objective fMRI or other investigations of effects on networks. This hypothesis motivated the search for dysfunctional nodes and networks in CFS.

## Materials and Methods

### Ethics

The protocol was approved by the Georgetown University Institutional Review Board (IRB 2013-0943 and 2015-0579) and listed in clinicaltrials.gov (NCT01291758 and NCT00810225). All clinical investigations were conducted according to the principles expressed in the Declaration of Helsinki.

### Subjects

Myalgic encephalomyelitis/chronic fatigue syndrome and healthy sedentary control subjects were recruited to these 4 day long in-patient studies in the Clinical Research Unit of the Georgetown–Howard Universities Center for Clinical and Translational Science. Subjects had history and physical examinations to ensure their inclusion by meeting Fukuda (Fukuda et al., 1994) and Carruthers Canadian Consensus Criteria for CFS (Carruthers et al., 2003; Carruthers, 2007), confirmation of sedentary lifestyle for control subjects (less than 40 min of aerobic activity per week), and exclusion because of serious medical or psychiatric conditions such as psychosis. History of posttraumatic stress disorder (PTSD) or depression were not exclusions unless the subject had been hospitalized in the past 5 years. Subjects completed a battery of questionnaires to assess self-reported complaints and symptoms (Rayhan et al., 2019).

### Orthostatic Status

The provocation component of the paradigm was two submaximal bicycle exercise stress tests that were performed 24 h apart. Subjects cycled for 25 min at 70% predicted maximum heart rate (HR) (220-patient’s age), followed by a climb to 85% maximum HR to reach anaerobic threshold (Garner et al., 2018). Many subjects could not reach this endpoint; exercise performance will be reported separately. Subjective complaints of post-exertional malaise, BOLD at rest and during the cognitive tests, and orthostatic status were compared between pre-exercise and post-exercise time periods.

Postural tachycardia was tested before and after exercise as a common element of the protocol (Rayhan et al., 2013; Garner et al., 2018; Garner and Baraniuk, 2019). Subjects rested supine for 5 min and had HR measured by continuous EKG monitor. After standing up, HR and blood pressure were measured every minute for 5 min. The incremental changes in HR between supine and standing (ΔHR) were calculated to identify episodes of postural tachycardia with ΔHR ≥ 30 bpm. The normal ΔHR was 12 ± 5 bpm (mean ± SD).

Stress Test Originated Phantom Perception (STOPP) was defined by ΔHR < 30 bpm at all times before and after exercise. STOPP subjects had normal cardiac responses when standing up and no change after exercise.

Stress Test Activated Reversible Tachycardia (START) was defined by having a normal ΔHR before exercise, but at least two measurements of postural tachycardia with ΔHR ≥ 30 bpm after exercise. This phenomenon was transient as ΔHR returned to normal 24–48 h following exercise.

Postural Orthostatic Tachycardia Syndrome (POTS) was defined by ΔHR ≥ 30 bpm at two or more measurements prior to exercise during the 5-min standing periods (Freeman et al., 2011). POTS criteria were met before and after exercise, and exercise did not exacerbate postural tachycardia in this subgroup indicating that POTS subjects were distinct from the START and STOPP groups.

### MRI Acquisition

Brain images were collected on a Siemens 3-Tesla Tim Trio scanner with a standard 12-channel head coil array. The fMRI data were attained using a T2^∗^-weighted gradient-echo planar imaging (EPI) during the resting state scan with the following imaging parameters: repetition time (TR) = 2500 ms, echo time (TE) = 30 ms, 90 degrees flip angle, FOV = 205mm^2^, matrix size = 64 × 64, number of slices = 47, voxel size = 3.2 mm^2^ isotropic. Runtime was 7 min with 168 total volumes acquired.

Structural scans were acquired with a 3D T1-weighted magnetization-prepared rapid acquisition with gradient echo (MPRAGE) pulse sequence with the following imaging parameters: TE = 2.52 ms, TR = 1900 ms, TI = 900 ms, FOV = 250 mm, 176 slices, slice resolution = 1.0 mm, voxel size 1 × 1 × 1 mm.

### Preprocessing of fMRI Data

Imaging data was preprocessed using FSL 5.0.11 (FMRIB, 2021; Smith et al., 2004), and Python 2.7. Preprocessing steps included removal of the first five images to allow for signal equilibration, brain extraction (FSL-BET), motion correction (FSL-MCFLIRT), interleaved slice-timing correction and linear co-registration (FSL-FLIRT) to subject specific T1-weighted MPRAGE plus standard MNI structural image. During motion correction (FSL-MCFLIRT), subject mean displacement was calculated. Subsequently, any subjects with relative mean displacement greater than 0.2 mm on either pre-exercise or post-exercise functional scans were excluded. This cutoff led to the exclusion of three ME/CFS participants from further analysis. Spatial smoothing of data was set at 5mm FWHM.

After standard preprocessing steps, FSL’s ICA-AROMA without temporal filtering was employed (Pruim et al., 2015a, b). We employed the non-aggressive option, which performed a partial component regression. ICA-AROMA carries out probabilistic ICA and does not require study-specific training prior to its use (i.e., manual classification of independent components). Outputs for each subject’s pre and post exercise rsfMRI data are interrogated utilizing four robust spatial and temporal features of motion-related components. The program then removed these components using an ordinary least squares regression. Temporal filtering can impose a high level of autocorrelation and ICA-AROMA is an robust alternative that preserves signal of interest and reproducibility or networks (Pruim et al., 2015a, b).

Final steps following ICA-AROMA included nuisance regression of structured noise (using subject-specific white matter, cerebrospinal fluid, and linear trends), and high-pass filtering (Pruim et al., 2015a, b). To aid in data preprocessing, multiple custom scripts were employed via command line.

### Network Analysis

Nodes and networks were identified by independent component analysis and MELODIC in FSL (MELODIC, 2021). A temporal concatenation approach was applied separately to pre-exercise and post-exercise scans by combining both ME/CFS and control patients (Beckmann et al., 2005). The method was successful in producing identifiable networks. However, the ability to compare across days was limited because group-ICA networks such as the DMN from pre-exercise and post-exercise had spatial differences. To provide comparable resting state networks across pre and post-exercise scans, we used a whole brain map template from a prior data set that generated 70 functional nodes (Smith et al., 2009). The nodal time-series for each subject’s pre and post exercise scans were extracted using the first step of the dual regression method in FSL using the template atlas (Smith et al., 2009). Following extraction of the 70 time-series for each scan, visual inspection identified 41 nodes that were artifacts and so were discarded.

The remaining 29 nodes were then entered into FSLnets and analyses was performed in MATLAB. Data from each node was entered to form matrices where individual and group networks were computed using the full-correlation option. Identified nodes/parcels were hierarchically clustered to reveal larger resting state networks. Visual inspection and comparison with well-defined resting state networks confirmed the clustered nodes and networks (Damoiseaux et al., 2006; Smith et al., 2009).

Because the spatial distribution of the network maps from the functional atlas was the same for both days, we analyzed the amplitude changes of the BOLD signal time-series between groups and across days. Changes within the amplitude may provide insight into dynamic exercise induced changes. Alterations of the BOLD amplitude have been associated with task-load during brain activation, pharmacological simulation, and illness (McKiernan et al., 2003; Kiviniemi et al., 2005; Vargas et al., 2013).

Amplitude strength for each node was calculated using the standard deviation of its associated time series (Li et al., 2017). Group differences were analyzed with two-tailed unpaired and paired *t*-tests across all analyzed nodes followed by Bonferroni correction for fifty-six comparisons (α = 0.05/56 = 0.000892) ANOVA and Tukey Honest Significant Difference for differences between groups and by paired Student’s *t*-test for exercise effects between days within groups in SPSS V.23. Functional connectivity was reported as Pearson’s correlation coefficient and α = 0.001 uncorrected (Clarke et al., 2019). To evaluate independent variables, general linear models used CFS status, orthostatic status, gender as fixed factors with age and BMI as continuous covariates. Multivariate general linear modeling was performed to assess the effects of Disease status (CFS vs. control) and Orthostatic status (STOPP vs. START vs. POTS).

## Results

### Demographics and Self-Reported Questionnaires

There were no significant differences in demographic variables between controls (*n* = 24) and ME/CFS subjects (*n* = 34) (Table 1). CFS severity questionnaire data revealed CFS subjects had significantly higher scores for fatigue, exertional exhaustion, unrefreshing sleep, and muscle pain (*P* < 0.001) (Baraniuk et al., 2013). Chalder fatigue total score (sum of 11 items, Σ11) and derived subscores were significantly higher in CFS than controls (*P* < 0.001) (Chalder et al., 1993; Cella and Chalder, 2010; Fong et al., 2015). No significant differences were found for the Center for Epidemiological Studies-Depression (CESD) domains (Vilagut et al., 2016).

**TABLE 1:** Demographic and questionnaire results.

	**Control**	**CFS**
N	24	34
Age	41.4 ± 17.9	46.9 ± 12.8
Females	10	25
STOPP	13	27
START	8	12
POTS	2	6
**CFS Severity Score**		
Fatigue	1.1 ± 1.1	3.4 ± 0.9*
Exertion	0.5 ± 1.0	3.5 ± 0.8*
Sleep	1.5 ± 1.4	3.2 ± 1.0*
Muscle pain	0.5 ± 0.9	2.6 ± 1.2*
Headaches	0.9 ± 1.2	2.1 ± 1.3
Joint pain	0.7 ± 1.0	1.8 ± 1.5
Sore throat	0.3 ± 0.6	1.1 ± 1.0
Lymph nodes	0.1 ± 0.5	0.9 ± 1.1
**Chalder Fatigue Score**		
Σ11 items	12.4 ± 5.2	22.4 ± 6.2*
Physical Σ8	9.1 ± 4.0	16.6 ± 5.0*
Mental Σ3	3.3 ± 1.5	5.8 ± 2.0*
Physical fatigue	3.8 ± 1.8	6.4 ± 2.0*
Less energy	4.3 ± 1.9	8.1 ± 2.6*
Mental fatigue	4.3 ± 1.8	7.9 ± 2.5*
**Center for Epidemiological Studies-Depression (CESD)**		
ΣCESD (0–60)	11.1 ± 11.8	17.9 ± 10.8
ΣCESD ≥ 16	27.3%	55.9%
Somatic factor	4.1 ± 4.6	8.9 ± 4.8
Depressed factor	2.8 ± 4.2	4.1 ± 4.2
Anhedonia factor	3.1 ± 3.0	4.3 ± 3.0
Interpersonal factor	1.0 ± 1.7	0.6 ± 1.2

*CFS severity, Chalder fatigue, and Center for Epidemiological Studies-Depression (CESD) questionnaires were compared between CFS and control groups.*

Mean ± SD. *P < 0.001 vs. control by *t*-test followed by Bonferroni correction for multiple comparisons.

### Group Average Hierarchical Clustering and Network Identification

**FIGURE 1:**
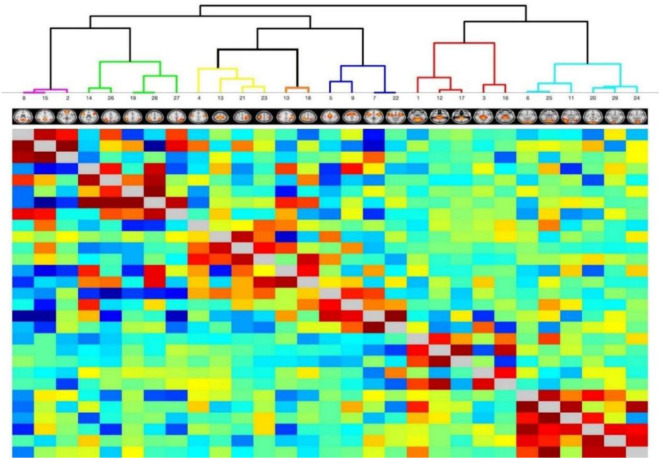
Hierarchical clustering heatmaps. Time series data identified 29 nodes in seven resting state networks as independent components (IC) using templates in FSLnets. Heatmap overlay is red-blue, where red signifies positive and blue signifies negative correlations, respectively. Clade color corresponds to parent resting state network. Purple clade = Default Mode Network (DMN). Green clade = Frontal Parietal Network (FPN). Yellow clade = Sensorimotor Network (SMN). Orange clade = Dorsal Attention Network (DAN). Blue clade = Salience Network (SAL). Red clade = Subcortical Network (SUB). Aqua clade = Visual Network (VIS). The insert figures show representative FSL probability maps to localize each node.

A total of 29 independent functional nodes extracted from the BOLD time series data were assessed using FSL by hierarchical clustering for network identification. The nodes were clustered into seven resting state networks: DMN, FPN, DAN, SMN, SAL, SUB, and VIS (Figure 1 and Table 2).

**TABLE 2:** Nodes and network abbreviations.

**IC**	**FSL**	**Node abbreviation**	**Approximate anatomical location**
**Default Mode Network (DMN)**			
8	0020	DMN_Prec	Precuneus
15	0030	DMN_PCC	Posterior cingulate cortex (PCC)
2	0006	DMN_mPFC	Medial prefrontal cortex (mPFC)
**Frontal Parietal Executive Control Network (FP)**			
14	0029	FP_DLPFC	Bilateral dorsolateral prefrontal cortex (DLPFC)
26	0055	FP_LPar	Left superior parietal lobule
19	0038	FP_biTPar	Bilateral temporoparietal region
28	0061	FP_biPar	Bilateral intraparietal sulcus/superior parietal lobule
27	0056	FP_RPar	Right superior parietal lobule
**Dorsal Attention Network (DAN)**			
10	0023	DAN_RIPS	Right intraparietal sulcus
18	0036	DAN_LIPS	Left intraparietal sulcus
**Salience Network (SAL)**			
5	0015	SAL_dACC	Dorsal anterior cingulate cortex
9	0021	SAL_SMA	Bilateral supplementary motor area
7	0019	SAL_aIns	Bilateral anterior insula
22	0048	SAL_mIns	Bilateral middle-posterior insula
**Subcortical (SUB)**			
1	0003	SUB_Vermis	Anterior cerebellum Vermis
2	0025	SUB_Midbrain	Midbrain
17	0032	SUB_Pons	Pons
3	0008	SUB_Thal	Thalamus
16	0031	SUB_vDien	Ventral diencephalon
**Sensorimotor Network (SM)**			
4	0009	SM_biM1	Bilateral prefrontal motor M1
13	0028	SM_biS1	Bilateral medial parietal sensory S1
21	0045	SM_RS2	Right parietal sensory S2
23	0050	SM_LS2	Left parietal sensory S2
**Visual Network (VIS)**			
6	0017	VIS0017	Bilateral parietal occipital sulcus
25	0054	VIS0054	Bilateral cuneus
11	0024	VIS0024	Bilateral lateral occipital gyrus
20	0041	VIS0041	Bilateral dorsal occipital lobe
29	0063	VIS0063	Left ventral occipital lobe
24	0053	VIS0053	Right ventral occipital lobe

*Seed nodes (FSL) and networks from independent component analysis (IC), abbreviations, and approximate anatomical locations are shown.*

### General Linear Models

Signal amplitude data were assessed by multivariate general linear model with CFS status, Orthostatic status and gender as fixed factors and age and body mass index (BMI) as independent co-variates. Disease status (CFS vs. control) was significant in models before and aft er exercise.

Preexercise control had significantly higher estimated marginal means than CFS for SAL_SMA, SM_RS2, SM_LS2, SUB_vDien, and VIS0063 after adjusting for the other variables (Table 3). Postexercise control had greater activation than CFS for salience (SAL_dACC, SAL_SMA, and SAL_mIns), sensorimotor SM_LS2, subcortical (SUB_Thal, SUB_Midbrain, SUB16vDien, and SUB_Pons), and visual (VIS0017, VIS0041, VIS0053, and VIS0063) network nodes. DMN_mPFC was significantly higher for CFS than control postexercise after adjusting for the other variables (Table 4).

**TABLE 3:** Prexercise GLM.

**Preexercise**	**Control**	**CFS**	**Univariate test significance**
DAN_RIPS	2.312 ± 0.320	2.062 ± 0.305	
DAN_LIPS	1.880 ± 0.284	1.595 ± 0.271	
DMN_mPFC	2.778 ± 0.458	2.271 ± 0.438	
DMN_Prec	2.853 ± 0.338	2.806 ± 0.323	
DMN_PCC	1.974 ± 0.196	1.818 ± 0.188	
FP_DLPFC	2.365 ± 0.353	1.931 ± 0.337	
FP_biTPar	2.163 ± 0.207	1.883 ± 0.198	
FP_LPar	1.982 ± 0.305	1.701 ± 0.292	
FP_RPar	1.963 ± 0.336	1.710 ± 0.321	
FP_biPar	1.931 ± 0.284	1.785 ± 0.271	
SAL_dACC	1.834 ± 0.264	1.554 ± 0.252	
SAL_aIns	2.120 ± 0.305	1.902 ± 0.292	
SAL_SMA	1.829 ± 0.318	1.317 ± 0.304	0.021
SAL_mIns	1.330 ± 0.200	1.095 ± 0.191	
SM_biM1	1.554 ± 0.280	1.298 ± 0.267	
SM_biS1	1.352 ± 0.232	1.038 ± 0.222	
SM_RS2	1.630 ± 0.303	1.120 ± 0.289	0.016
SM_LS2	1.468 ± 0.265	0.988 ± 0.253	0.01
SUB_Vermis	1.325 ± 0.224	1.052 ± 0.214	
SUB_Thal	1.067 ± 0.136	0.902 ± 0.130	
SUB_Midbrain	0.971 ± 0.141	0.809 ± 0.135	
SUB_vDien	0.889 ± 0.106	0.703 ± 0.101	0.012
SUB_Pons	1.109 ± 0.223	0.899 ± 0.214	
VIS0017	1.480 ± 0.207	1.280 ± 0.197	
VIS0054	1.359 ± 0.252	1.249 ± 0.240	
VIS0024	1.864 ± 0.246	1.579 ± 0.236	
VIS0041	1.633 ± 0.269	1.494 ± 0.256	
VIS0053	1.799 ± 0.300	1.428 ± 0.287	
VIS0063	1.580 ± 0.222	1.275 ± 0.212	0.047

*Preexercise multivariate GLM with CFS status, Orthostatic status and gender as fixed factors, and age and BMI as independent variables was significant (Wilks’ lambda = 0.202, *p* = 0.036, Partial Eta Squared = 0.798). Orthostatic status, BMI and gender were not significant covariates. Estimated marginal mean ± 95%CI. BOLD signal amplitudes were higher in control than CFS by univariate significance.*

**TABLE 4:** Postexercise GLM.

**Postexercise**	**Control**	**CFS**	**Univariate test significance**	**Partial Eta squared**	**Magnitude**
DAN_RIPS	2.170 ± 0.310	2.160 ± 0.297			
DAN_LIPS	1.832 ± 0.248	1.548 ± 0.238			
DMN_mPFC	1.928 ± 0.404	2.855 ± 0.387	0.001	0.204	CFS > SC
DMN_Prec	2.836 ± 0.319	2.683 ± 0.305			
DMN_PCC	1.899 ± 0.207	1.822 ± 0.197			
FP_DLPFC	2.245 ± 0.265	1.892 ± 0.254			
FP_biTPar	2.029 ± 0.231	1.955 ± 0.221			
FP_LPar	1.873 ± 0.233	1.734 ± 0.223			
FP_RPar	1.747 ± 0.222	1.664 ± 0.212			
FP_biPar	1.881 ± 0.221	1.726 ± 0.212			
SAL_dACC	1.799 ± 0.181	1.553 ± 0.174	0.05	0.083	SC > CFS
SAL_aIns	2.010 ± 0.205	1.854 ± 0.195			
SAL_SMA	1.634 ± 0.184	1.355 ± 0.176	0.029	0.101	SC > CFS
SAL_mIns	1.341 ± 0.127	1.126 ± 0.122	0.016	0.123	SC > CFS
SM_biM1	1.584 ± 0.266	1.378 ± 0.254			
SM_biS1	1.272 ± 0.228	1.134 ± 0.218			
SM_RS2	1.667 ± 0.301	1.286 ± 0.288			
SM_LS2	1.593 ± 0.248	1.083 ± 0.238	0.004	0.171	SC > CFS
SUB_Vermis	1.385 ± 0.210	1.116 ± 0.200			
SUB_Thal	1.023 ± 0.103	0.862 ± 0.098	0.025	0.107	SC > CFS
SUB_Midbrain	1.076 ± 0.123	0.847 ± 0.118	0.009	0.144	SC > CFS
SUB_vDien	0.858 ± 0.086	0.737 ± 0.082	0.043	0.088	SC > CFS
SUB_Pons	1.103 ± 0.151	0.820 ± 0.144	0.008	0.147	SC > CFS
VIS0017	1.553 ± 0.209	1.210 ± 0.200	0.019	0.117	SC > CFS
VIS0054	1.529 ± 0.237	1.080 ± 0.227	0.007	0.149	SC > CFS
VIS0024	1.911 ± 0.319	1.494 ± 0.305			
VIS0041	1.736 ± 0.236	1.319 ± 0.225	0.012	0.132	SC > CFS
VIS0053	1.950 ± 0.240	1.494 ± 0.23	0.007	0.15	SC > CFS
VIS0063	1.635 ± 0.185	1.359 ± 0.177	0.032	0.098	SC > CFS

*Postexercise multivariate GLM with CFS status (CFS vs. Sedentary Control), Orthostatic status and gender as fixed factors, and age and BMI as independent variables was significant (Wilks’ lambda = 0.201, *p* = 0.035, Partial Eta Squared = 0.799). Orthostatic status and gender were not significant covariates. Estimated marginal mean ± 95%CI. Univariate significance.*

Age was significant in univariate analysis for many nodes before and after exercise. The interactions of age, Disease status and gender were evaluated as fixed factors. Using the preexercise data, age was significant for FP_LPar, FP_RPar, SAL_aIns, SAL_dACC, both DAN nodes, DMN_Prec, and VIS0053. However, only FP_RPar was significant for CFS status (estimated marginal means for Control 2.181 ± 0.136 and CFS 1.701 ± 0.117, mean ± 95%CI, *p* = 0.016) in this model. Postexercise confirmed significant age effects for FP_LPar, FP_RPar, SAL_aIns, and VIS0053 plus FP_biPar, but only DMN_mPFC was significantly associated with CFS after accounting for age and gender.

Orthostatic status and BMI were not significant before or after exercise.

The incremental differences between postexercise and preexercise (Δ) were significantly different in two nodes. DMN_mPFC was larger for CFS than control postexercise, while the difference for VIS0017 was larger in control than CFS (Table 5).

**TABLE 5:** GLM for incremental changes.

**Δ**	**SC**	**CFS**	**Univariate test significance**	**Partial Eta Squared**	**Magnitude**
DMN_mPFC	−0.850 ± 0.448	0.583 ± 0.428	0	0.333	CFS > SC
VIS0017	0.170 ± 0.232	−0.169 ± 0.223	0.036	0.094	SC > CFS

*Multivariate GLM for exercise induced changes (Δ = post-exercise minus pre-exercise) in BOLD with CFS status, Orthostatic status and gender as fixed factors, and age and BMI as independent variables was significant (Wilks’ lambda = 0.201, *p* = 0.035, Partial Eta Squared = 0.799).*

*Orthostatic status and gender were not significant. Estimated marginal mean ± 95%CI. Univariate significance.*

### Analysis of Variance

The data were compared by ANOVA after taking the significant variables into account. The GLM results showed that BOLD was significantly different by Disease status, exercise day, and age in univariate analyses. The demographics found different proportions by gender in control and CFS. Orthostatic status and BMI were not significant variables. Therefore, signal amplitude data were regressed for age and gender and the differences between CFS and control were assessed on the preexercise and postexercise days (Table 6). Several general trends were identified that were compared to those found by GLM.

**TABLE 6:** ANOVA.

**Network ROI**	**Pre-exercise**		**Post-exercise**		**Pre-exercise**	**Post-exercise**
	**Control**	**CFS**	**Control**	**CFS**		
**Reciprocal exercise effects in sedentary control and CFS**						
DMN_mPFC	2.761 ± 1.010	2.329 ± 0.865	2.100 ± 0.740	2.965 ± 0.776		Post CFS > SC *p* = 0.001
Exercise effect (paired *t*-test)					SC: Pre > Post *p* = 0.0029	CFS: Post > Pre *p* = 0.00036
					Pre SC > CFS	Post SC > CFS
**Persistent deficit in CFS preexercise and postexercise**						
SAL_mIns	1.358 ± 0.594	1.041 ± 0.249	1.328 ± 0.325	1.058 ± 0.263	0.008	0.031
SUB_Midbrain	0.948 ± 0.362	0.739 ± 0.205	1.023 ± 0.378	0.778 ± 0.187	0.031	0.008
SUB_Pons	1.102 ± 0.563	0.822 ± 0.346	1.074 ± 0.469	0.787 ± 0.206	0.046	0.038
VIS0063	1.597 ± 0.650	1.207 ± 0.329	1.585 ± 0.560	1.227 ± 0.403	0.015	0.031
**Baseline preexercise CFS deficit**						
DAN_LIPS	1.944 ± 0.796	1.474 ± 0.399	1.855 ± 0.621	1.526 ± 0.418	0.01	
DMN_PCC	2.019 ± 0.484	1.713 ± 0.330	1.931 ± 0.457	1.704 ± 0.467	0.045	
FP_biTPar	2.191 ± 0.563	1.771 ± 0.368	2.130 ± 0.614	1.923 ± 0.432	0.009	
FP_RPar	2.139 ± 0.815	1.673 ± 0.520	1.815 ± 0.535	1.652 ± 0.434	0.015	
SAL_SMA	1.959 ± 0.880	1.339 ± 0.435	1.639 ± 0.397	1.360 ± 0.313	0	
SMN_RS2	1.644 ± 0.772	1.164 ± 0.398	1.587 ± 0.645	1.289 ± 0.554	0.014	
SMN_LS2	1.482 ± 0.788	1.007 ± 0.321	1.459 ± 0.675	1.117 ± 0.420	0.009	
SUB_Thal	1.021 ± 0.361	0.832 ± 0.189	1.015 ± 0.248	0.850 ± 0.209	0.028	
SUB_vDien	0.831 ± 0.298	0.670 ± 0.124	0.836 ± 0.217	0.710 ± 0.163	0.017	
**Exercise induced deficit in CFS**						
VIS0017	1.315 ± 0.718	1.057 ± 0.409	1.434 ± 0.714	1.018 ± 0.381		0.027
VIS0054	1.462 ± 0.574	1.156 ± 0.349	1.538 ± 0.577	1.186 ± 0.335		0.022
VIS0041	1.622 ± 0.743	1.306 ± 0.445	1.632 ± 0.693	1.231 ± 0.420		0.045
VIS0053	1.782 ± 0.965	1.416 ± 0.416	1.838 ± 0.774	1.375 ± 0.421		0.041
**No differences between CFS and control before or after exercise**						
DAN_RIPS	2.321 ± 0.864	1.870 ± 0.516	2.275 ± 0.826	1.949 ± 0.555		
FP_DLPFC	2.373 ± 0.798	1.978 ± 0.608	2.238 ± 0.655	1.895 ± 0.450		
FP_biPar	2.055 ± 0.691	1.690 ± 0.497	1.985 ± 0.642	1.666 ± 0.404		
SAL_dACC	1.843 ± 0.696	1.520 ± 0.365	1.841 ± 0.516	1.577 ± 0.293		
SAL_aIns	2.131 ± 0.748	1.777 ± 0.507	2.077 ± 0.508	1.756 ± 0.405		
SUB_Vermis	1.271 ± 0.568	0.966 ± 0.337	1.355 ± 0.532	1.054 ± 0.353		
VIS0024	1.757 ± 0.778	1.429 ± 0.404	1.823 ± 0.795	1.443 ± 0.586		
DMN_Prec	2.821 ± 0.746	2.767 ± 0.633	2.856 ± 0.816	2.727 ± 0.567		
FP_LPar	2.069 ± 0.832	1.741 ± 0.447	1.921 ± 0.641	1.723 ± 0.373		
SMN_biM1	1.547 ± 0.708	1.251 ± 0.409	1.559 ± 0.506	1.342 ± 0.538		
SMN_biS1	1.355 ± 0.609	1.058 ± 0.345	1.276 ± 0.464	1.166 ± 0.442		

*Node amplitude strengths were the average of the signal over the 7 min resting scan time series for each node and subject. Multivariate general linear modeling (GLM) of the strengths in each node utilized Disease status, Orthostatic status and gender as fixed factors and age and BMI as co-variates. Nodes that were significantly different based on Disease status (CFS vs. control) after accounting for the other variables were indicated by “GLM.” Orthostatic status and BMI were not significant variables in the models and were removed from further models. Because (a) age was a significant variable for many nodes by univariate analysis and (b) the groups had unequal gender proportions, the raw strengths were regressed against age and gender. Significant differences between control and CFS on preexercise and postexercise days were calculated using ANOVA with Tukey Honest Significant Difference to correct for multiple comparisons. Node amplitude strength was reported as mean ± SD. Significant Tukey results were reported for Preexercise Control > CFS, Postexercise Control > CFS, and CFS > Control. Exercise effects on each group were assessed by paired *t*-tests.*

The average BOLD amplitude from all measurements before exercise was significantly higher for control (1.749 ± 0.854, mean ± SD) than CFS (1.410 ± 0.616, *p* = 10^–20^ by two-tailed unpaired *t*-test comparing all regions between all control and CFS subjects). After exercise, control (1.691 ± 0.724) was again significantly higher than CFS (1.450 ± 0.664, *p* = 10^–12^). This suggested an overarching disease effect with lower cerebral blood flow in CFS than controls, and an exercise effect with significantly higher activation preexercise than postexercise in both control (*p* = 0.0017, two-tailed paired *t*-test) and CFS (*p* = 0.0070, two-tailed paired *t*-test).

DMN_mPFC was the most interesting node because of the reciprocal effects of exercise in CFS compared to control. The two groups were equivalent prior to exercise. BOLD in sedentary control was greater in preexercise than postexercise indicating a relative reduction in the activity in DMN_mPFC on the second day. In contrast, CFS had increased activation of DMN_mPFC in the resting state following the exercise stressor. As a result, CFS had significantly greater BOLD than control after exercise. This was consistent with the GLM of incremental changes (Table 5).

Four regions had persistently lower BOLD in CFS compared to control before and after exercise (Table 6) indicating a persistent deficit that did not change with provocation. The regions were SAL_mIns, SUB_Midbrain, SUB_Pons, and VIS0063.

CFS had lower BOLD than control at baseline for DAN_LIPS, DMN_PCC, FP_biTPar, FP_RPar, SAL_SMA, SM_rS2, SM_LS2, SUB_Thal and SUB_vDien. Estimated marginal means were not significantly different after exercise.

Exercise caused reduced BOLD in CFS compared to postexercise controls in VIS0017, VIS0057, VIS0041, and VIS0053.

Other nodes were not different between control and CFS or with exercise indicating that they were not affected by disease status or provocation.

**FIGURE 2:**
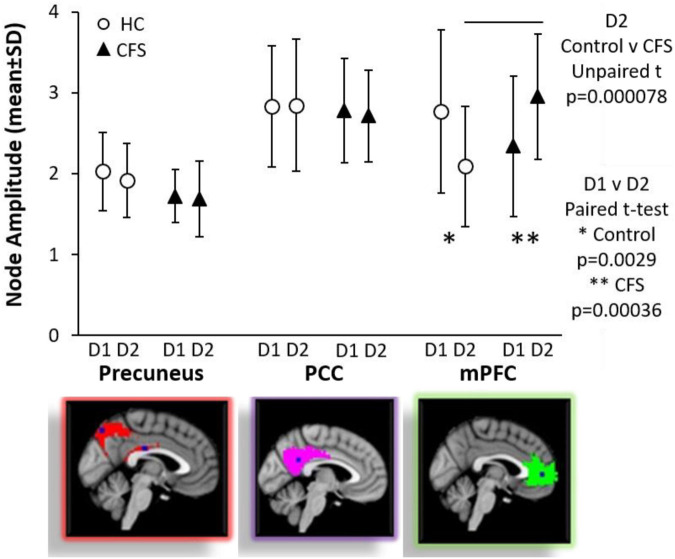
Default mode network nodes. Node amplitude strengths were compared by ANOVA and Tukey Honest Significant Difference for differences between groups and by paired Student’s *t*-test for exercise effects between days within groups. DMN_PCC (red in top figure on right) and DMN_Prec (magenta in middle figure on right) were equivalent between control and CFS and between preexercise and postexercise scans. In contrast, exercise had significant effects on the anterior node in the DMN_mPFC (green in the bottom figure on the right). Node amplitude strengths were equivalent for CFS and control preexercise. Exercise caused a significant decrease in control (^∗^*p* = 0.0029 by paired test), but a significant increase for CFS (^∗∗^*p* = 0.00036 by paired test). As a result of the dynamic changes, CFS had significantly higher signal than control postexercise (line above error bars, *p* = 0.000078). Mean ± SD.

There was general agreement between the results of the GLMs using raw amplitude data and ANOVA performed after the data were regressed versus age and gender. The most important was the reciprocal changes in DMN_mPFC after exercise (Figure 2).

### Connectivity

Connectivity between nodes was assessed by Pearson correlations using the age and gender regressed data from the CFS and control groups on each study day. Each set of regressed amplitudes was demeaned by the respective group averages (subject minus average) for each node. Net BOLD signals from each node were correlated using two-tailed Pearson tests. There were no significant negative correlations.

**FIGURE 3:**
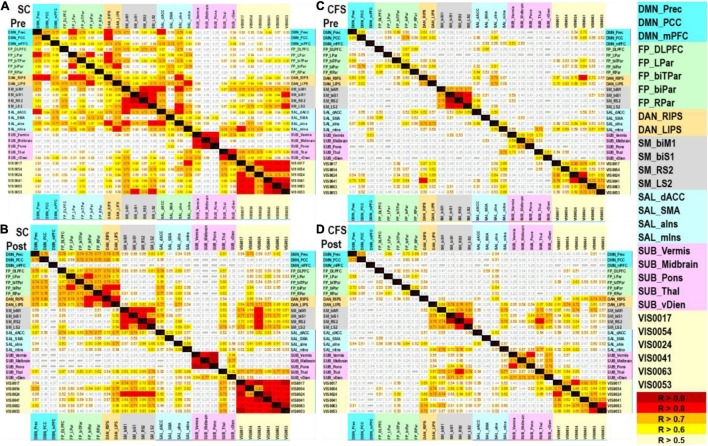
Pearson correlation matrices. Age and gender regressed BOLD amplitude data were correlated for **(A)** sedentary control preexercise, **(B)** sedentary control postexercise, **(C)** CFS preexercise, and **(D)** CFS postexercise. Correlations are shown with *R* > 0.5 (light yellow), *R* > 0.6 (yellow), *R* > 0.7 (orange), *R* > 0.8 (red), and *R* > 0.9 (dark red) that were all *p* < 0.001 uncorrected. There were no significant negative correlations. Networks and nodes were highlighted in color on the right: DMN cyan, FPN green, DAN orange, SMN gray, SAL blue, SUB pink, and VIS yellow.

Prexercise control showed correlations within each network but particularly Sensorimotor (yellow edge), Visual (light blue edge), and Salience (red edge) (Figure 3). DMN_mPFC was significantly correlated (*R* > 0.7, *p* < 0.001 uncorrected) with DMN_Prec, FP_DLPFC, DAN_RIPS, DAN_LIPS, SAL_dACC, SAL_SMA, and SAL_aIns.

Postexercise control was more highly correlated within each network than preexercise suggesting improved efficiency when resting in the scanner on the second day. DMN_mPFC had zero significant correlations postexercise (*R* < 0.5).

Preexercise CFS was markedly different as there were significant correlations with *R* > 0.8 only within the sensorimotor and visual networks. DMN_mPFC was correlated with SUB_Vermis (*R* = 0.633).

Exercise modestly increased the number of significant correlations in CFS, but this number was smaller than control following exercise. DMN_mPFC in CFS had no correlations larger than 0.3 despite the increased activation of this node after exercise.

The age and gender regressed BOLD values had no significant correlations with measures of fatigue, pain, tenderness, depression, and other subjective symptoms (all *R* < 0.3, data not shown).

## Discussion

The remarkable discordant finding in CFS was the exercise – induced signal increase within the medial prefrontal cortex of the DMN. This change may be a functional indicator of post-exertional malaise that is a requirement for the diagnosis of CFS. The DMN is a collection of brain regions with correlated activation during rest when there is no externally oriented task to be performed (Raichle, 2015). DMN regions become deactivated when switching from rest to task performance as other regions simultaneously become coordinated in specific task networks. As a result, the relative activation levels of DMN and task network nodes are anti-correlated. The medial prefrontal cortex (mPFC) is the anterior hub of the DMN (Müller et al., 2020). Generally, it is recognized that the DMN is involved in self-referential though, mind-wandering, and autobiographic memory retrieval (Gusnard et al., 2001; Mason et al., 2007; Spreng and Grady, 2010). The mPFC also contributes to other aspects of cognition such as social awarenes, affective processing, and goal-directed behavior (Schilbach et al., 2008; Spreng and Grady, 2010; Roy et al., 2012). The relative activation of the mPFC caused by exercise in CFS may represent a decoupling from the posterior nodes of the DMN and loss of regulatory input. Thus, exercise – induced uncoupling may be a pathological consequence and a cognitive biomarker of post-exertional malaise. If so, this finding has significant potential to become a criterion for disease diagnosis.

All regions except mPFC of the DMN tended to have lower resting state BOLD signal in CFS compared to control before and after exercise (Table 6). There were three general patterns. First, CFS had a pre-exercise deficit in parietal, SMA, thalamus, and ventral diencephalon regions, but after exercise the differential narrowed and became insignificant because of a general decline in control with unchanged values in CFS. Second, visual regions showed the opposite trend as they were equivalent prior to exercise. However, after exercise the combination of a slight increase in signal for controls and no change in CFS led to significant exercise-induced deficits. The last pattern was persistent deficits in CFS for Midbrain, Pons, and insular nodes pre and post exercise. The insula, midbrain and pons are hubs for interoception, autonomic regulation, pain, sleep, and motor regulation (Bracha, 2004; Critchley et al., 2004; Blanchard et al., 2011; Buhle et al., 2013; Nakatomi et al., 2014; Blanchard, 2017; Franklin, 2019). Deficits in these regions may correlate to the persistent visceral symptoms experienced in CFS. The general trend for lower BOLD signal at rest in CFS compared to controls is consistent with the general reduction in cerebral blood flow and reports of orthostatic intolerance that are recurrent findings in CFS (Razumovsky et al., 2003; Yoshiuchi, 2006; Pardini et al., 2010; Tajima et al., 2010; Biswal et al., 2011; Stewart et al., 2011; Ocon et al., 2012; Medow et al., 2014; Gay et al., 2016; Finkelmeyer et al., 2018; van Campen et al., 2020a, b, c).

Chronic fatigue syndrome had blunted activity throughout the cerebrum compared to controls before and after exercise except for within the mPFC. Increased signal in the mPFC has been associated with the fatigue in TBI and during processing of fatigue as a sensation (Pardini et al., 2010; Tajima et al., 2010). A similar finding of elevated anterior DMN activation during a low cognitive load task following exercise was also found in Gulf War Illness (GWI) (Rayhan et al., 2019). GWI is a chronic disease affecting approximately 25% of the veterans who served in the 1991 Persian Gulf War (Fukuda et al., 1998; Steele, 2000; White et al., 2016). CFS and GWI have similar symptoms of fatigue, cognitive dysfunction and post-exertional malaise (Rayhan et al., 2019; Washington et al., 2020a, b).

Age is an important confounder for DMN function and uncoupling between DMN nodes can be a result of aging (Damoiseaux et al., 2008; Dennis and Thompson, 2014; Puttaert et al., 2020; Kavroulakis et al., 2021). However, aging does not explain the increase in BOLD in the mPFC seen in CFS as age was regressed within the GLM and ANOVA analyses (Figure 2).

Limitations of the study are findings may only be limited to our resting state and submaximal exercise provocation protocol. Replicative studies will help provide further validate the results. BOLD signals were processed extensively to account for age, gender and motion, and were reported as the standard deviations of the nodal amplitude changes. Data analysis by ANOVA of age and gender regressed data and GLM gave comparable outcomes. It is possible that other variables related to fatigue, regulation of pain or interoceptive signaling, affect, anxiety, autonomic dysfunction, and/or molecular mechanisms of neurovascular coupling may have had significant effects on our findings. Functions related to the diencephalon, brainstem, vermis, and insula that were significantly activated here may also provide additional covariates for future evaluation. Comparison of anterior DMN activation between CFS and GWI was only qualitative but future studies including both groups may provide additional results into exercise-induced dysfunction and the role of the mPFC in fatigue processing. Comparisons to other cognitive tasks such as Stroop and Flanker are likely to provide additional information about baseline and post-exertional cognitive processing. A standard template was used to generate the initial seed regions for the analyses. It is possible those actual regions of activation and deactivation did not conform to these seeds, and that the apparent levels were contaminated by BOLD activity bleeding over from adjacent brain regions. Despite this possibility, the anterior DMN node had reciprocal changes after exercise in CFS versus controls.

Future analysis using ICA nodes generated from native subject data may provide a clearer picture to the exercise-induced dysfunction and network interaction occurring in CFS patients. Future studies will explore the effectiveness of potential therapies by assessing whether they can prevent the post-exertional increase in activation within the anterior DMN (mPFC) in CFS. Samples sizes were relatively large; the mean and standard deviations provide effect size estimates to power future confirmatory studies of anterior DMN dysfunction in post-exertional symptom exacerbation in CFS.

## Conclusion

The important exercise effect was the differential change in anterior DMN node activation following exercise. CFS had significantly increased activation while control had a decrease in activation compared to the preexercise day. CFS is defined clinically by postexertional malaise and cognitive difficulties (Fukuda et al., 1994; Carruthers et al., 2003; Carruthers, 2007; Institute of Medicine, 2015). The postexertional increase in BOLD activation in the medial prefrontal cortex may represent a biomarker for CFS that indicates loss of focus or excessive mind wandering during the resting state compared to controls. Alternative explanations for reduced cognition would be a decrease in salience, dorsal attention, and frontoparietal executive functioning but these were not detected in the resting state.

## Data Availability Statement

The original contributions presented in the study are included in the article/supplementary material, further inquiries can be directed to the corresponding author.

## Ethics Statement

The studies involving human participants were reviewed and approved by Georgetown University. The patients/participants provided their written informed consent to participate in this study.

## Author Contributions

JB obtained the funding. JB and RR performed the research program, data analysis, and wrote the manuscript. Both authors contributed to the article and approved the submitted version.

## Conflict of Interest

The authors declare that the research was conducted in the absence of any commercial or financial relationships that could be construed as a potential conflict of interest.

## Publisher’s Note

All claims expressed in this article are solely those of the authors and do not necessarily represent those of their affiliated organizations, or those of the publisher, the editors and the reviewers. Any product that may be evaluated in this article, or claim that may be made by its manufacturer, is not guaranteed or endorsed by the publisher.

## References

[B1] Baraniuk J. N. Adewuyi O. Merck S. J. Ali M. Ravindran M. K. Timbol C. R. (2013). A chronic fatigue syndrome (CFS) severity score based on case designation criteria. *Am. J. Transl. Res.* 5 53–68. 23390566 PMC3560481

[B2] Beckmann C. F. DeLuca M. Devlin J. T. Smith S. M. (2005). Investigations into resting-state connectivity using independent component analysis. *Philos. Trans. R. Soc. Lond. B Biol. Sci.* 360 1001–1013. 10.1098/rstb.2005.1634 16087444 PMC1854918

[B3] Biswal B. Kunwar P. Natelson B. H. (2011). Cerebral blood flow is reduced in chronic fatigue syndrome as assessed by arterial spin labeling. *J. Neurol. Sci.* 301 9–11. 21167506 10.1016/j.jns.2010.11.018 PMC3139492

[B4] Blanchard D. C. (2017). Translating dynamic defense patterns from rodents to people. *Neurosci. Biobehav. Rev.* 76 22–28. 10.1016/j.neubiorev.2016.11.001 28434585

[B5] Blanchard D. C. Griebel G. Pobbe R. Blanchard R. J. (2011). Risk assessment as an evolved threat detection and analysis process. *Neurosci. Biobehav. Rev.* 35 991–998. 10.1016/j.neubiorev.2010.10.016 21056591

[B6] Bracha H. S. (2004). Freeze, flight, fight, fright, faint: adaptationist perspectives on the acute stress response spectrum. *CNS Spectr.* 9 679–685. 10.1017/s1092852900001954 15337864

[B7] Buhle J. T. Kober H. Ochsner K. N. Mende-Siedlecki P. Weber J. Hughes B. L. (2013). Common representation of pain and negative emotion in the midbrain periaqueductal gray. *Soc. Cogn. Affect. Neurosci.* 8 609–616. 10.1093/scan/nss038 22446299 PMC3739905

[B8] Carruthers B. M. (2007). Definitions and aetiology of myalgic encephalomyelitis: how the Canadian consensus clinical definition of myalgic encephalomyelitis works. *J. Clin. Pathol.* 60 117–119. 10.1136/jcp.2006.042754 16935963 PMC1860613

[B9] Carruthers B. M. Jain A. K. De Meirleir K. L. Peterson D. L. Klimas N. G. Lerner A. M. (2003). Myalgic encephalomyelitis/chronic fatigue syndrome. *J. Chronic Fatigue Syndr.* 11 7–115. 10.1300/J092v11n01_02

[B10] Carruthers B. M. van de Sande M. I. De Meirleir K. L. Klimas N. G. Broderick G. Mitchell T. (2011). Myalgic encephalomyelitis: international consensus criteria. *J. Intern. Med.* 270 327–338. 10.1111/j.1365-2796.2011.02428.x 21777306 PMC3427890

[B11] Cella M. Chalder T. (2010). Measuring fatigue in clinical and community settings. *J. Psychosom. Res.* 69 17–22. 10.1016/j.jpsychores.2009.10.007 20630259

[B12] Chalder T. Berelowitz G. Pawlikowska T. Watts L. Wessely S. Wright D. (1993). Development of a fatigue scale. *J. Psychosom. Res.* 37 147–153. 10.1016/0022-3999(93)90081-P 8463991

[B13] Clarke T. Jamieson J. D. Malone P. Rayhan R. U. Washington S. VanMeter J. W. (2019). Connectivity differences between Gulf War Illness (GWI) phenotypes during a test of attention. *PLoS One* 14:e0226481. 10.1371/journal.pone.0226481 31891592 PMC6938369

[B14] Critchley H. D. Wiens S. Rotshtein P. Ohman A. Dolan R. J. (2004). Neural systems supporting interoceptive awareness. *Nat. Neurosci.* 7 189–195. 10.1038/nn1176 14730305

[B15] Damoiseaux J. S. Beckmann C. F. Arigita E. J. S. Barkhof F. Scheltens P. Stam C. J. (2008). Reduced resting-state brain activity in the “default network” in normal aging. *Cereb. Cortex* 18 1856–1864. 10.1093/cercor/bhm207 18063564

[B16] Damoiseaux J. S. Rombouts S. A. R. B. Barkhof F. Scheltens P. Stam C. J. Smith S. M. (2006). Consistent resting-state networks across healthy subjects. *Proc. Natl. Acad. Sci. U.S.A.* 103 13848–13853. 10.1073/pnas.0601417103 16945915 PMC1564249

[B17] Dennis E. L. Thompson P. M. (2014). Functional brain connectivity using fMRI in aging and Alzheimer’s disease. *Neuropsychol. Rev.* 24 49–62. 10.1007/s11065-014-9249-6 24562737 PMC4109887

[B18] Esposito F. Bertolino A. Scarabino T. Latorre V. Blasi G. Popolizio T. (2006). Independent component model of the default-mode brain function: assessing the impact of active thinking. *Brain Res. Bull.* 70 263–269. 10.1016/j.brainresbull.2006.06.012 17027761

[B19] Finkelmeyer A. He J. Maclachlan L. Blamire A. M. Newton J. L. (2018). Intracranial compliance is associated with symptoms of orthostatic intolerance in chronic fatigue syndrome. *PLoS One* 13:e0200068. 10.1371/journal.pone.0200068 29969498 PMC6029803

[B20] Fong T. C. T. Chan J. S. M. Chan C. L. W. Ho R. T. H. Ziea E. T. C. Wong V. C. W. (2015). Psychometric properties of the Chalder Fatigue scale revisited: an exploratory structural equation modeling approach. *Qual. Life Res.* 24 2273–2278. 10.1007/s11136-015-0944-4 25688039 PMC4529874

[B21] Fox M. D. Snyder A. Z. Vincent J. L. Corbetta M. Van Essen D. C. Raichle M. E. (2005). From the cover: the human brain is intrinsically organized into dynamic, anticorrelated functional networks. *Proc. Natl. Acad. Sci. U.S.A.* 102 9673–9678. 10.1073/pnas.0504136102 15976020 PMC1157105

[B22] Franklin T. B. (2019). Recent Advancements Surrounding the Role of the Periaqueductal Gray in Predators and Prey. *Front. Behav. Neurosci.* 13:60. 10.3389/fnbeh.2019.00060 31133827 PMC6524621

[B23] Freeman R. Wieling W. Axelrod F. B. Benditt D. G. Benarroch E. Biaggioni I. (2011). Consensus statement on the definition of orthostatic hypotension, neurally mediated syncope and the postural tachycardia syndrome. *Clin. Auton. Res.* 21 69–72. 10.1007/s10286-011-0119-5 21431947

[B24] FMRIB (2021). *FMRIB Software Library v6.0. Created by the Analysis Group.* Oxford: FMRIB.

[B25] Fukuda K. Nisenbaum R. Stewart G. Thompson W. W. Robin L. Washko R. M. (1998). Chronic multisymptom illness affecting Air Force veterans of the Gulf War. *J. Am. Med. Assoc.* 280 981–988. 10.1001/jama.280.11.981 9749480

[B26] Fukuda K. Straus S. E. Hickie I. Sharpe M. C. Dobbins J. G. Komaroff A. (1994). The chronic fatigue syndrome: a comprehensive approach to its definition and study. *Ann. Intern. Med.* 121 953–959. 10.7326/0003-4819-121-12-199412150-00009 7978722

[B27] Garner R. S. Rayhan R. U. Baraniuk J. N. (2018). Verification of exercise-induced transient postural tachycardia phenotype in Gulf War Illness. *Am. J. Transl. Res.* 10 3254–3264. 30416666 PMC6220213

[B28] Garner R. Baraniuk J. N. (2019). Orthostatic intolerance in chronic fatigue syndrome. *J. Transl. Med.* 17:185. 10.1186/s12967-019-1935-y 31159884 PMC6547462

[B29] Gay C. W. Robinson M. E. Lai S. O’Shea A. Craggs J. G. Price D. D. (2016). Abnormal resting-state functional connectivity in patients with chronic fatigue syndrome: results of seed and data-driven analyses. *Brain Connect.* 6 48–56. 10.1089/brain.2015.0366 26449441 PMC4744887

[B30] Gusnard D. A. Akbudak E. Shulman G. L. Raichle M. E. (2001). Medial prefrontal cortex and self-referential mental activity: relation to a default mode of brain function. *Proc. Natl. Acad. Sci. U.S.A.* 98 4259–4264. 10.1073/pnas.071043098 11259662 PMC31213

[B31] Institute of Medicine (2015). *Beyond Myalgic Encephalomyelitis/Chronic Fatigue Syndrome.* Washington, DC: National Academies Press, 10.17226/19012 25695122

[B32] Kavroulakis E. Simos N. J. Maris T. G. Zaganas I. Panagiotakis S. Papadaki E. (2021). Evidence of age-related hemodynamic and functional connectivity impairment: a resting state fMRI study. *Front. Neurol.* 12:633500. 10.3389/fneur.2021.633500 33833727 PMC8021915

[B33] Kiviniemi V. Ruohonen J. Tervonen O. (2005). Separation of physiological very low frequency fluctuation from aliasing by switched sampling interval fMRI scans. *Magn. Reson. Imaging* 23 41–46. 10.1016/j.mri.2004.09.005 15733787

[B34] Li H. Nickerson L. D. Nichols T. E. Gao J.-H. (2017). Comparison of a non-stationary voxelation-corrected cluster-size test with TFCE for group-Level MRI inference. *Hum. Brain Mapp.* 38 1269–1280. 10.1002/hbm.23453 27785843 PMC6866890

[B35] Mason M. F. Norton M. I. Van Horn JD Wegner D. M. Grafton S. T. Macrae C. N. (2007). Wandering minds: the default network and stimulus-independent thought. *Science* 315 393–395. 10.1126/science.1131295 17234951 PMC1821121

[B36] McKiernan K. A. Kaufman J. N. Kucera-Thompson J. Binder J. R. (2003). A parametric manipulation of factors affecting task-induced deactivation in functional neuroimaging. *J. Cogn. Neurosci.* 15 394–408. 10.1162/089892903321593117 12729491

[B37] Medow M. S. Sood S. Messer Z. Dzogbeta S. Terilli C. Stewart J. M. (2014). Phenylephrine alteration of cerebral blood flow during orthostasis: effect on n-back performance in chronic fatigue syndrome. *J. Appl. Physiol.* 117 1157–1164. 10.1152/japplphysiol.00527.2014 25277740 PMC4233252

[B38] MELODIC (2021). *FMRIB Software Library v6.0. Created by the Analysis Group.* Oxford: FMRIB.

[B39] Müller N. C. J. Dresler M. Janzen G. Beckmann C. F. Fernández G. Kohn N. (2020). Medial prefrontal decoupling from the default mode network benefits memory. *Neuroimage* 210:116543. 10.1016/j.neuroimage.2020.116543 31940475

[B40] Nakatomi Y. Mizuno K. Ishii A. Wada Y. Tanaka M. Tazawa S. (2014). Neuroinflammation in patients with chronic fatigue syndrome/myalgic encephalomyelitis: an11C-(R)-PK11195 PET study. *J. Nucl. Med.* 55 945–950. 10.2967/jnumed.113.131045 24665088

[B41] National Institute for Health and Care Excellence [NICE] (2021). *Myalgic Encephalomyelitis (or Encephalopathy)/Chronic Fatigue Syndrome: Diagnosis and Management. NICE Guideline [NG206].* Available online at: https://www.nice.org.uk/guidance/ng206 (accessed October 29, 2021). 35438859

[B42] Ocon A. J. Messer Z. R. Medow M. S. Stewart J. M. (2012). Increasing orthostatic stress impairs neurocognitive functioning in chronic fatigue syndrome with postural tachycardia syndrome. *Clin. Sci. (Lon.)* 122 227–238. 10.1042/CS20110241 21919887 PMC3368269

[B43] Pardini M. Krueger F. Raymont V. Grafman J. (2010). Ventromedial prefrontal cortex modulates fatigue after penetrating traumatic brain injury. *Neurology* 9 749–754. 10.1212/WNL.0b013e3181d25b6b PMC2836872 20194914

[B44] Pruim R. H. R. Mennes M. Buitelaar J. K. Beckmann C. F. (2015a). Evaluation of ICA-AROMA and alternative strategies for motion artifact removal in resting state fMRI. *Neuroimage* 112 278–287. 10.1016/j.neuroimage.2015.02.063 25770990

[B45] Pruim R. H. R. Mennes M. van Rooij D. Llera A. Buitelaar J. K. Beckmann C. F. (2015b). ICA-AROMA: a robust ICA-based strategy for removing motion artifacts from fMRI data. *Neuroimage* 112 267–277. 10.1016/j.neuroimage.2015.02.064 25770991

[B46] Puttaert D. Coquelet N. Wens V. Peigneux P. Fery P. Rovai A. (2020). Alterations in resting-state network dynamics along the Alzheimer’s disease continuum. *Sci. Rep.* 10:21990. 10.1038/s41598-020-76201-3 33319785 PMC7738511

[B47] Raichle M. E. (2015). The Brain’s default mode network. *Annu. Rev. Neurosci.* 8 433–447. 10.1146/annurev-neuro-071013-014030 25938726

[B48] Rayhan R. U. Stevens B. W. Raksit M. P. Ripple J. A. Timbol C. R. Adewuyi O. (2013). Exercise challenge in gulf war illness reveals two subgroups with altered brain structure and function. *PLoS One* 8:e63903. 10.1371/journal.pone.0063903 23798990 PMC3683000

[B49] Rayhan R. U. Washington S. D. Garner R. Zajur K. Martinez Addiego F. Vanmeter J. W. (2019). Exercise challenge alters default mode network dynamics in Gulf War Illness. *BMC Neurosci.* 20:7. 10.1186/s12868-019-0488-6 30791869 PMC6385399

[B50] Razumovsky A. Y. DeBusk K. Calkins H. Snader S. Lucas K. E. Vyas P. (2003). Cerebral and systemic hemodynamics changes during upright tilt in chronic fatigue syndrome. *Neuroimaging* 13 57–67. 10.1152/ajpheart.00994.2011 12593133

[B51] Roy M. Shohamy D. Wager T. D. (2012). Ventromedial prefrontal-subcortical systems and the generation of affective meaning ventromedial prefrontal cortical involvement across psychological. *Trends Cogn. Sci.* 16 147–156. 10.1016/j.tics.2012.01.005.Ventromedial 22310704 PMC3318966

[B52] Schilbach L. Eickhoff S. B. Rotarska-Jagiela A. Fink G. R. Vogeley K. (2008). Minds at rest? Social cognition as the default mode of cognizing and its putative relationship to the “default system” of the brain. *Conscious. Cogn.* 17 457–467. 10.1016/j.concog.2008.03.013 18434197

[B53] Smith S. M. Fox P. T. Miller K. L. Glahn D. C. Fox P. M. Mackay C. E. (2009). Correspondence of the brain’s functional architecture during activation and rest. *Proc. Natl. Acad. Sci. U.S.A.* 106 13040–13045. 10.1073/pnas.0905267106 19620724 PMC2722273

[B54] Smith S. M. Jenkinson M. Woolrich M. W. Beckmann C. F. Behrens T. E. J. Johansen-Berg H. (2004). Advances in functional and structural MR image analysis and implementation as FSL. *Neuroimage* 23 S208–S219. 10.1016/j.neuroimage.2004.07.051 15501092

[B55] Spreng R. N. Grady C. L. (2010). Patterns of brain activity supporting autobiographical memory, prospection, and theory of mind, and their relationship to the default mode network. *J. Cogn. Neurosci.* 22 1112–1123. 10.1162/jocn.2009.21282 19580387

[B56] Steele L. (2000). Prevalence and patterns of Gulf War illness in Kansas veterans: association of symptoms with characteristics of person, place, and time of military service. *Am. J. Epidemiol.* 152 992–1002. 10.1093/aje/152.10.992 11092441

[B57] Stewart J. M. Medow M. S. Messer Z. R. Baugham I. L. Terilli C. Ocon A. J. (2011). Postural neurocognitive and neuronal activated cerebral blood flow deficits in young chronic fatigue syndrome patients with postural tachycardia syndrome. *Am. J. Physiol. Heart Circ. Physiol.* 302 1185–1194. 10.1152/ajpheart.00994.2011 PMC3311460 22180650

[B58] Tajima S. Yamaoto S. Tanaka M. Kataoka Y. Masoa I. Yoshikawa E. (2010). Medial orbitofrontal cortex is associated with fatigue sensation. *Neurol. Res. Int.* 2010:671421. 10.1155/2010/671421 21188225 PMC3003967

[B59] Uddin L. Q. Clare Kelly A. M. Biswal B. B. Xavier Castellanos F. Milham M. P. (2009). Functional connectivity of default mode network components: correlation, anticorrelation, and causality. *Hum. Brain Mapp.* 30 625–637. 10.1002/hbm.20531 18219617 PMC3654104

[B60] van Campen C. L. M. C. Rowe P. C. Verheugt F. W. A. Visser F. C. (2020a). Cognitive function declines following orthostatic stress in adults with Myalgic Encephalomyelitis/Chronic Fatigue syndrome (ME/CFS). *Front. Neurosci.* 14:688. 10.3389/fnins.2020.00688 32670016 PMC7332734

[B61] van Campen C. L. M. C. Rowe P. C. Verheugt F. W. A. Visser F. C. (2020b). Orthostatic stress testing in myalgic encephalomyelitis/chronic fatigue syndrome patients with or without concomitant fibromyalgia: effects on pressure pain thresholds and temporal summation. *Clin. Exp. Rheumatol.* 39(Suppl 130) 39–47. 10.55563/clinexprheumatol/1qj9zu 32940215

[B62] van Campen C. L. M. C. Verheugt F. W. A. Rowe P. C. Visser F. C. (2020c). Cerebral blood flow is reduced in ME/CFS during head-up tilt testing even in the absence of hypotension or tachycardia: a quantitative, controlled study using Doppler echography. *Clin. Neurophysiol. Pract.* 5 50–58. 10.1016/j.cnp.2020.01.003 32140630 PMC7044650

[B63] Vanasse T. J. Fox P. T. Fox P. M. Cauda F. Costa T. Smith S. M. (2021). Brain pathology recapitulates physiology: a network meta-analysis. *Commun. Biol.* 4 301. 10.1038/s42003-021-01832-9 33686216 PMC7940476

[B64] Vargas C. ópez-Jaramillo C. L. Vieta E. (2013). A systematic literature review of resting state network-functional MRI in bipolar disorder. *J. Affect. Disord.* 150 727–735. 10.1016/j.jad.2013.05.083 23830141

[B65] Vilagut G. Forero C. G. Barbaglia G. Alonso J. (2016). Screening for depression in the general population with the center for epidemiologic studies depression (CES-D): a systematic review with meta-analysis. *PLoS One* 11:e0155431. 10.1371/journal.pone.0155431 27182821 PMC4868329

[B66] Washington S. D. Rayhan R. U. Garner R. Provenzano D. Zajur K. Addiego F. M. (2020a). Exercise alters brain activation in gulf war illness and Myalgic Encephalomyelitis/Chronic Fatigue Syndrome. *Brain Commun.* 2:fcaa070. 10.1093/braincomms/fcaa070 32954325 PMC7425336

[B67] Washington S. D. Rayhan R. U. Garner R. Provenzano D. Zajur K. Addiego F. M. (2020b). Exercise alters cerebellar and cortical activity related to working memory in phenotypes of Gulf War Illness. *Brain Commun.* 2:fcaa070. 10.1093/braincomms/fcz039 32025659 PMC6989731

[B68] White R. F. Steele L. O’Callaghan J. P. Sullivan K. Binns J. H. Golomb B. A. (2016). Recent research on Gulf War illness and other health problems in veterans of the 1991 Gulf War: effects of toxicant exposures during deployment. *Cortex* 74 449–475. 10.1016/j.cortex.2015.08.022 26493934 PMC4724528

[B69] Yoshiuchi K. (2006). Patients with chronic fatigue syndrome have reduced absolute cortical blood flow. *Clin. Physiol. Funct. Imaging* 26 83–86. 10.1111/j.1475-097X.2006.00649.x 16494597

